# Inexplicable Inefficiency of Avian Molt? Insights from an Opportunistically Breeding Arid-Zone Species, *Lichenostomus penicillatus*

**DOI:** 10.1371/journal.pone.0016230

**Published:** 2011-02-02

**Authors:** Bethany J. Hoye, William A. Buttemer

**Affiliations:** 1 School of Biological Sciences, Institute for Conservation Biology, University of Wollongong, Wollongong, Australia; 2 Department of Animal Ecology, Netherlands Institute for Ecology (NIOO-KNAW), Wageningen, The Netherlands; 3 Centre for Integrative Ecology, School of Life and Environmental Sciences, Deakin University, Waurn Ponds, Australia; University of Queensland, Australia

## Abstract

The majority of bird species studied to date have molt schedules that are not concurrent with other energy demanding life history stages, an outcome assumed to arise from energetic trade-offs. Empirical studies reveal that molt is one of the most energetically demanding and perplexingly inefficient growth processes measured. Furthermore, small birds, which have the highest mass-specific basal metabolic rates (BMR_m_), have the highest costs of molt per gram of feathers produced. However, many small passerines, including white-plumed honeyeaters (WPHE; *Lichenostomus penicillatus*), breed in response to resource availability at any time of year, and do so without interrupting their annual molt. We examined the energetic cost of molt in WPHE by quantifying weekly changes in minimum resting metabolic rate (RMR_min_) during a natural-molt period in 7 wild-caught birds. We also measured the energetic cost of feather replacement in a second group of WPHEs that we forced to replace an additional 25% of their plumage at the start of their natural molt period. Energy expenditure during natural molt revealed an energy conversion efficiency of just 6.9% (±0.57) close to values reported for similar-sized birds from more predictable north-temperate environments. Maximum increases in RMR_min_ during the molt of WPHE, at 82% (±5.59) above individual pre-molt levels, were some of the highest yet reported. Yet RMR_min_ maxima during molt were not coincident with the peak period of feather replacement in naturally molting or plucked birds. Given the tight relationship between molt efficiency and mass-specific metabolic rate in all species studied to date, regardless of life-history pattern (Efficiency (%)  = 35.720•10^−0.494*BMRm*^; r^2^ = 0.944; p = <0.0001), there appears to be concomitant physiological costs entrained in the molt period that is not directly due to feather replacement. Despite these high total expenditures, the protracted molt period of WPHE significantly reduces these added costs on a daily basis.

## Introduction

Variations in life-history patterns are thought to represent a given species' maximization of lifetime inclusive fitness within a particular environment [1], [2]. The channeling of available resources during a specific time period into a single, resource-demanding activity is the most widely recognized, and perhaps most common pattern of resource partitioning [2]. Accordingly, widespread theoretical and empirical investigation reveals that the annual cycle of birds living in north temperate and high latitudes is characterized by pronounced temporal separation of the most intense phases of molt, breeding and migration, with little or no overlap between these activities at the individual level [3]–[7]. Indeed, birds that initiate molt during their final stages of breeding (in either naturally or experimentally induced late breeding pairs) often experience considerable fitness costs as parents [8–11, but see 12], as do their offspring [13]–[15]. Partitioning of molt from other life history stages is therefore assumed to be an adaptation that minimizes physiological stress while maximizing the allocation of productive energy [16].

Maintenance of the aerodynamic, insulative and signaling functions of avian plumage is of paramount importance and requires annual feather replacement in most bird species. During a complete molt, a bird must synthesize almost one-quarter of its total body protein in the form of feathers and other epidermal structures [17], [18]. This places a high demand on energy and nutrients, especially protein [19]. Therefore, detailed knowledge of the relative energy requirements of molt is integral for gaining insight into avian life history strategies.

Feather production costs, estimated from measurement of increases in basal metabolic rate throughout the molt period, show up to ten-fold variation between species. Small passerines, such as bluethroats (*Luscinia s. svecica*; 17 g), and redpolls (*Carduelis f. flammea*; 13 g) expend between 862 and 709 kJ·g dry feathers^–1^ [20], respectively, compared with 69 and 116 kJ·g dry feathers^–1^ for the kookaburra (*Dacelo novaeguineae*; 335 g; [21]) and the long-eared owl (*Asio otus*; 280 g; [22]), respectively. Much of this size-related variation in molt cost is thought to be a consequence of mass-related differences in metabolic rate, which predicts that the smallest birds, with the highest mass-specific metabolic rates (BMR_m_), will have the highest molt costs and hence lowest energy conversion efficiencies in feather production [20], [23], [24].

It is difficult, however, to reconcile predictions of high molt costs for small passerines with the observation that molt and breeding are often coincident in opportunistically breeding small passerine species living in unpredictable habitats [7], [25]–[27]. This is especially true of Australia's old-endemic passerines living in arid zones. Because erratic rainfall and periodic droughts are characteristic of this habitat [28], resource availability does not correspond with annual variation in photoperiod, in marked contrast with many temperate locations [29]. Consequently, residents of arid zones initiate breeding opportunistically, in order to match finite periods of favorable environmental conditions [26], [30], [31]. Molt in these birds, however, shows surprisingly little inter-annual variation in its schedule [26], despite vast irregularities in available resources and reproductive status [32]. Clearly, in these birds, breeding and molt are not temporally incompatible.

The coincidence of molt/breeding overlap in individuals of some species, but not in others, provokes the question: Is molt an inherently costly and inefficient process, or are costs dependent upon other life history characteristics? We examined these questions by measuring molt costs in the White-plumed honeyeater (WPHE; *Lichenostomus penicillatus*), an old-endemic Australian species that includes populations inhabiting the arid zone. These populations display a flexible breeding schedule that corresponds with unpredictable flushes of lerps, the larvae of a psylid insect associated with the River Redgums (*Eucalyptus camaldulensis*) on which adults forage [33]. In stark contrast, molt follows a regular schedule regardless of environmental conditions, and molt/breeding overlap is a regular occurrence [26], [32]. We aimed to see if their ability to molt and breed concurrently was associated with lower costs of molt. We also investigated whether the reported energetic inefficiency of feather production during molt was due to feather replacement *per se* by measuring the energetic consequences of feather replacement following artificial removal of 25% of feathers by mass in a separate group of honeyeaters. We found that energetic expenditure during natural molt in WPHE was surprisingly similar to expectations based on birds from more predictable north-temperate environments, but that these increases in energy expenditure were not coincident with the peak period of feather replacement in naturally molting or plucked birds.

## Methods

### Ethics statement

The animals used in this study were captured under license from the New South Wales National Parks and Wildlife service (S11320). All experimental procedures were carried out under approval from the University of Wollongong Animal Ethics Committee (AE04/12), in accordance with the Australian Code of Practice for the Care and Use of Animals for Scientific Purposes. Feathers were plucked while birds were under methoxyflurane anesthesia, and all efforts were made to minimize any suffering throughout the study. Birds were released at the site of capture following completion of the study.

### Study animals

White-plumed honeyeaters (WPHE) were caught in mist nets at Fowler's Gap Arid-Zone Research Station, New South Wales (31°S, 142°E) in August, and brought to the University of Wollongong (34°25′S, 150°54′E), where they were held in constant temperature (25°C) rooms under natural photoperiod. Birds were housed two per cage (ca. 40×60×60 cm), and were provided with commercial honeyeater food (Wombaroo Pty. Ltd. – min crude protein 13%) and water *ad libitum*.

### Metabolic measurements

Birds were held for two weeks to habituate to captive conditions before determining pre-molt basal metabolic rate (BMR). By definition, BMR represents minimum metabolic rates while animals are post-absorptive, in the rest-phase of their daily cycle; and exposed to thermoneutral temperatures. Furthermore, BMR requires there to be an absence of energetically demanding activities, such as growth, reproduction, or molt. We therefore refer to metabolic rate measurements taken under these conditions during molt as minimum resting metabolic rate (RMR_min_). Each individual's RMR_min_ was then measured weekly until the completion of feather regrowth. Birds were fasted for approximately 2 h before being removed from cages between 16:30 and 18:00, weighed, and then placed in individual 4-L respirometers. Respirometers were held in a constant temperature cabinet overnight, set at 33°C (± 1°C), verified to be thermally neutral for this species [personal obs., 34]. Air provided to the respirometers passed through a desiccant (Drierite) and was regulated at 400 mL/min by calibrated mass-flow controllers (Tylan Corp.). Respirometers were removed from the cabinet between 08:00 and 09:00, at which time birds were reweighed and assessed for molt (see below). Oxygen consumption rates (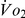; ml/min) were evaluated by comparing measurements of O_2_ content of the inlet and outlet air for each chamber using an Oxzilla oxygen analyzer (Sable Systems), sampled every 5 seconds. Each bird's BMR was measured sequentially for 15 min out of every 36 min throughout the night. Because basal metabolism is characterized by extended periods of stable 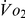 [35], RMR_min_ was calculated as the mean of the two lowest five-minute running averages of 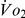 measured, usually between 02:00 and 05:00. Metabolic rates (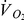) were converted to kJ·d^−1^ using the conversion 1 L O_2_  =  20.08 kJ [36]. All reported values of oxygen consumption represent STP conditions, and volume effects on gas concentrations due to inequalities in respiratory quotient have been addressed using an equation appropriate to the measurement system [37]:
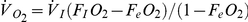



where 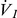
represents air flow rate corrected to STP conditions, *F_I_O_2_* represents fractional O_2_ content in inlet air after removing CO_2_ and H_2_O, and *F_e_O_2_* represents fractional O_2_ content in excurrent chamber air after removing CO_2_ and H_2_O.

### Energetic cost of molt

Five individuals were euthanized prior to the onset of molt and all feathers plucked, divided into 10 regions: head, neck, dorsal body, ventral body, dorsal covert, ventral covert, cloacal, primary, secondary and rectrices. The feathers were then dried overnight at 80°C and weighed to the nearest mg. The total energy content of the plumage was calculated assuming that dry feathers have an energy content of 26.4 kJ·g^−1^ [19]. The total cost of molt in each individual (below) was taken as the total increase in BMR above pre-molt levels over the molt period, plus the energy contained in the new plumage.

### Energetic cost of feather replacement

A further 24 birds were randomly assigned to one of two treatments. “Plucked” birds (n = 12), once anaesthetized (methoxyflurane), had 25% of their body feathers, by weight, removed from each of the seven body regions outlined above, along with two feathers each from the secondaries (s6 and s7) and primaries (p7 and p8) of each wing and two centre rectrices. Plucking took place in the first week of October. Control (“sham plucked”) birds (n = 12) were also anaesthetized, but then simply handled for one minute and allowed to recover. Metabolic rates and feather growth rates (mm·day^–1^ [38]) were recorded weekly for each individual until they had fully replaced their artificially removed feathers (6 weeks). These measurements were continued for the duration of natural molt in the sham-plucked birds. The natural molt data are based on measurements of seven control birds due to the death of three birds following an equipment failure, and the inability of two birds to fully adjust to captive conditions (as evidenced by large fluctuations in body mass).

### Statistical Analyses

Differences in individual metabolic rates between different time periods were analyzed using repeated measures ANOVA using JMP 7 (SAS). Comparison of metabolic rates, metabolic increases and feather growth rates between natural molting and plucked individuals were conducted using one-way ANOVA, again using JMP 7. All values given below represent mean (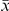) ± 1 S.E. unless otherwise stated.

### Interspecific variation in molt cost

We compiled data on the cost of molt per gram of feathers replaced for all species studied using respirometry (indirect calorimetry), and used these to calculate conversion efficiency (energy contained within the plumage as a percentage of energy expended during its growth; Table S1). Using a tree pruned from Hackett *et al*. [39] and the regressionv2.m package [40] in MATLAB (R2007b), we first fitted the best of several models of evolution to the efficiency of feather production data, including species values (ordinary least squares, no phylogenetic signal), Brownian motion (phylogenetic generalized least squares), Ornstein–Uhlenbeck process (OU, drift about a fitness peak) or with branch lengths transformed using Pagel's λ parameter. We then tested for a relationship between these traits and metabolic intensity (BMR_m_). The OU and Pagel's λ fitting procedures calculate branch length transformation parameters, d for the OU process that is a function of time and λ for Pagel's transformation, which is constant across the tree, by restricted maximum likelihood (REML). Values close to 1 indicate a significant phylogenetic signal, whereas values close to 0 suggest the trait in question is independent of phylogeny.

## Results

### Molt phenology

White-plumed honeyeaters initiated molt on 13 October (±6.0 days, range 27 September - 14 November; n = 7). This appeared similar to the onset of molt in the wild, with birds at Fowler's Gap Research Station displaying the same range of molt stage as those in captivity in Wollongong in early January (personal obs.). Feather replacement lasted for 168 days (± 5.21; range  =  143–196 days), from the loss of the innermost primary until the completion of feather replacement in all tracts.

### Energetic cost of molt

**Figure 1:**
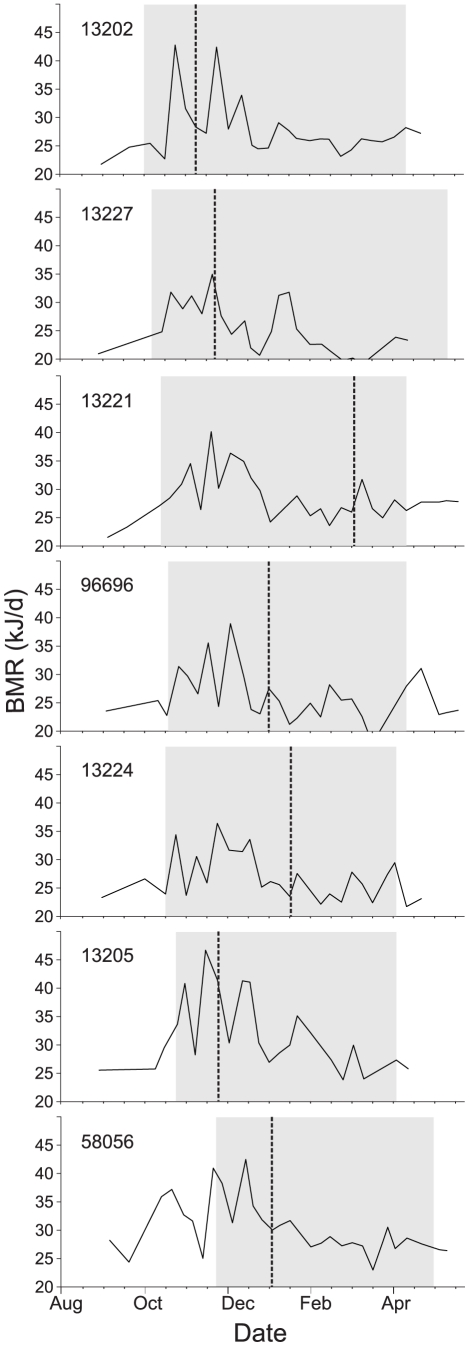
Minimum resting metabolic rate of individual White-plumed honeyeaters during molt. Minimum resting metabolic rate (RMR_min_ in kJ·d^−1^) of 7 captive White-plumed Honeyeaters preceding, during, and following the period of natural molt. Numbers indicate individual identifiers. Grey shaded areas represent a given individual's molt period, dashed vertical lines represent the individual's period of peak feather replacement.

Pre-molt BMR, measured at least 30 days prior to the loss of the first primary, averaged 23.0 kJ·d^−1^ (± 0.65, n = 7). An individual's highest RMR_min_ during the molt period was recorded 30 days (± 3.7) after molt onset, and was 18.43 kJ·day^−1^ (± 1.14) above their pre-molt BMR, representing an 82% (± 5.59) increase. Each individual's highest RMR_min_ value during molt was significantly greater than both pre-molt BMR and RMR_min_ measured at the time of their most intense feather replacement (*F_2_*_,*5*_ = 97.41; *P*<0.0005; Table 1). The pattern of RMR_min_ throughout the molt period, however, did not gradually rise preceding an individual's maxima, nor show a graded decline thereafter. Instead, each bird's RMR_min_, both during and outside the molt period, appeared to oscillate stochastically, with multiple peaks and troughs evident. Importantly, the pattern and size of these oscillations did not directly correspond with molt stage (Figure 1).

**Table 1:** Minimum resting metabolic rates during molt and replacement of plucked feathers.

	Natural molt		Plucked	
Pre molt	23.04	±0.65	23.41	±0.54
Peak feather replacement	30.77	±5.41	29.51	±1.22
Maximum RMR_min_	40.84∧	±4.22	39.26	±1.14

Minimum resting metabolic rates (kJ·d^−1^) at various stages of feather replacement in captive White-plumed honeyeaters undergoing natural molt (n = 7) compared to conspecifics undergoing natural molt while simultaneously replacing an additional 25% of their plumage that was plucked during the experiment (n = 12; means ± SE).

∧ indicates significantly higher values within treatments (p<0.05); there were no significant differences between treatments at any stage.

The most intense period of feather replacement occurred 56 (± 13.7) days after molt onset, at which time metabolic rates were not significantly higher than an individual's pre-molt BMR (Table 1). Furthermore, individuals yet to molt displayed very similar RMR_min_ profiles with respect to calendar date to their molting conspecifics, irrespective of their molt schedule (Figure 1).

Because the post-molt BMR averaged 13% (±5.7) higher than pre-molt levels (*t* = 2.46; *d.f.* = 6; *P* = 0.049), the BMR “baseline” used to judge molt costs on a given date was based on an interpolation between pre- and post-molt BMR measurements for each individual. These values were not adjusted for body mass as variation in individual mass accounted for less than 5% of the overall variation in RMR_min_ during molt (*F_1_*_,*186*_ = 8.93; *r^2^* = 0.046; *P* = 0.0032). Integrating the daily increases in metabolic rate above this baseline throughout each bird's molt period produced an average energetic cost of feather synthesis of 453.4 kJ·bird^−1^ (±66.6). The energetic cost of molt (C_f_; kJ.g^−1^ dry feathers) represents the sum of synthesis costs and the energy contained in the replacement plumage. Given an average total plumage mass of 1.28 g measured in non-molting birds and an energy content of 26.4 kJ·g^−1^ [19], the C_f_ in WPHE averaged 380.6 kJ·g dry feathers^−1^ (±49.3). Thus, the energy content of the newly synthesized plumage represented just 6.9% (±0.57) of the energy expended for feather renewal.

### Energetic cost of feather replacement

There was no significant difference in natural molt initiation date between plucked and sham-plucked (control) WPHE (*F_1_*_,*21*_ = 0.713; *P* = 0.682). Primary feather growth rates were linear until feathers approached their full length, after which time growth rates slowed. Growth rates for the linear part of plucked feather re-growth were indistinguishable from those of corresponding primaries in sham-plucked (naturally molting) WPHEs (*P*>0.200; Table 2). Plucked birds also grew their naturally molted primaries (one and two) at rates that were statistically indistinguishable from sham-plucked birds (*P*>0.200; Table 2).

**Table 2:** Primary feather growth rates during molt and replacement of plucked feathers.

Feather	Natural molt		Plucked		*t*	*d.f.*	*p*
Primary 1	2.70	±0.13	2.74	±0.16	−0.732	10	0.732
Primary 2	2.83	±0.13	2.97	±0.18	−1.087	9	0.293
Primary 7	2.65	±0.07	2.57	±0.07	0.876	12	0.393
Primary 8	2.60	±0.19	2.35	±0.18	1.277	12	0.232

Feather growth rates (mm/d ± S.E.) of selected primaries in captive White-plumed honeyeaters undergoing natural molt compared to conspecifics undergoing natural molt while simultaneously replacing an additional 25% of their plumage that was plucked during the experiment.

The metabolic rate profiles of plucked birds did not differ significantly from those of naturally molting conspecifics in relation to calendar date, despite the overlap of naturally molted and plucked feather regrowth in the 10 plucked individuals. The RMR_min_ of plucked birds prior to molt, during the peak phase of feather regrowth, and at the individual maxima were not significantly different to RMR_min_ measured contemporaneously in sham plucked (naturally molting) birds (*P*>0.05 Table 1).

### Interspecific variation in molt cost

**Figure 2:**
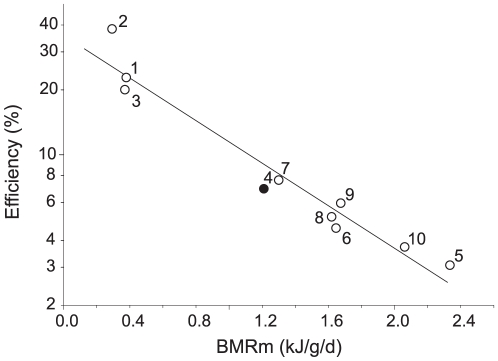
Feather production efficiency during molt. Feather production efficiency (%; 100·kJ content·[kJ expended + content]^−1^) during natural molt in relation to mass specific BMR (kJ·g^−1^·d^−1^) measured using indirect calorimetry for different species of birds. Filled circle represents our white-plumed honeyeater (*Lichenostomus penicillatus;* 4), unfilled circles represent long-eared owl (*Asio otus*; 1), kookaburra (*Dacelo novaeguineae*; 2), European kestrel (*Falco tinnunculus*; 3), bluethroat (*Luscinia s. Svecica*; 5), European stonechat (*Saxicola torquata rubicula*; 6), East African stonechat (*Saxicola torquata axillaries*; 7), white-crowned sparrow (*Zonotrichia leucophrys gambelii*; 8), chaffinch (*Fringilla coelebs*; 9), and redpoll (*Carduelis f. Flammea*; 10). Values and sources in Table S1.

Our estimate of the energetic cost (and hence efficiency) of molt in WPHE adds to a growing body of evidence suggesting that these traits differ markedly between certain species (Table S1). The efficiency of energy conversion to new feathers during molt showed no relationship to either relative feather mass (*P* = 0.36) or molt rate (*P* = 0.06). Efficiency was, however, highly correlated with metabolic intensity (mass-specific basal metabolic rate, BMR_m_, kJ.g^−1^.d^−1^; *r^2^* = 0.944, *P* = <0.0001; Figure 2) such that:
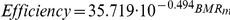



Phylogenetic regression revealed a strong phylogenetic signal in the relationship between BMR_m_ and conversion efficiency during molt (Table S2), yet the relationship between BMR_m_ and efficiency remained highly significant (*r^2^* = 0.884). Models based on phylogenetic relationships alone (null model) were equally parsimonious as the metabolic intensity model, however this is not unexpected given that efficiency was almost synonymous with BMR_m_ (*r^2^* = 0.944).

The average daily cost of molt (total cost (kJ)/duration (days)) of WPHE was equivalent to 13% of their pre-molt BMR; the lowest daily molt cost of all species studied (Table S1). There was no apparent phylogenetic signal in the interspecies variation in these average daily costs (3).

## Discussion

White-plumed honeyeaters experience a considerable increase in RMR_min_ during the population's molt period, despite living in an environment with unpredictable resource availability and often experiencing molt/breeding overlap. Maximum increases in RMR_min_ during a molt cycle have been shown to be as little as 10% of pre-molt levels in large birds such as the 280 g long-eared owl [22], and as high as 111% and 106% in bluethroats (17 g) and redpolls (13 g), respectively [20]. White-plumed honeyeaters showed some of the highest reported increases in metabolic rate, averaging 82% above individual pre-molt levels (Table S1).

Increases in RMR_min_ were not, however, coincident with the temporal stage of feather replacement in either naturally molting or plucked birds. Surprisingly, individual WPHE reached their metabolic maxima relatively synchronously with respect to calendar date, regardless of their molt stage (Figure 1). Further, RMR_min_ maxima were no higher in the plucked birds than in naturally molting conspecifics (Table 1), despite replacing an additional 25% of their plumage at the same rate as naturally molting birds (Table 2). White-crowned sparrows have also been shown to replace substantial amounts of plucked feathers without experiencing a rise in RMR_min_ [41], demonstrating that feather replacement *per se* can not explain the increased energy demands measured during the molt period. Moreover, weekly measures of RMR_min_ in individual WPHE did not follow a graded rise and a steady decline about the period of feather replacement maxima. Large RMR_min_ fluctuations were measured periodically, suggesting that coincident physiological processes are of a cyclical nature. Importantly, such week-to-week fluctuations would be overlooked in studies with less frequent metabolic measurements.

Efficiency of energy conversion during molt (energy contained in the feathers produced as a proportion of energy expended) is enigmatically low in all birds studied and, with the exception of kookaburras [21], remains the most inefficient form of protein conversion described in vertebrates [42]. The energetic cost of the molt period in WPHE was remarkably similar to values reported for other small passerines from more predictable north-temperate environments [e.g. 23]. Furthermore, the WPHE values are consistent with accumulating evidence that metabolic rate increases during molt scale to a species' metabolic intensity (BMR_m_: kJ·g^−1^·d^−1^) consistently across all species examined [20], [23], [24]. This predicts that the smallest birds with the highest mass-specific metabolic rates will have the highest costs, and hence the lowest conversion efficiencies (Figure 2). Although there appears to be a phylogenetic component to this relationship, interpretation of the influence of phylogeny on the costs entrained in the molt period is hindered by the complete lack of data from small (i.e. high metabolic intensity) non-passerine species and large (i.e. low metabolic intensity) passerine species. Given the tight relationship between molt efficiency and mass-specific metabolic rate in all species studied to date, regardless of life-history pattern, there appears to be underlying costs entrained in the molt period that are proportional to metabolic intensity, but are not due to feather replacement *per se*. Efficiency may, therefore, be an artifact of the assumption that metabolic increases during molt are solely fueling feather synthesis.

There is ample evidence that molting birds undergo extensive physiological changes beyond the production of feathers alone, including plasma volume expansion [43], [44], and organ hypertrophy [45]. In addition, there is increasing evidence that immune processes are altered during the molt period, with evidence of energetically- and nutritionally-demanding acute-phase and inflammatory responses being down-regulated during this period of the annual cycle (Table 3; [46]–[48]). This would reduce the competition between immunity and molt for nutritional resources, thus permitting birds to maintain consistent feather growth rates when immune challenged [49] and, perhaps more importantly, to ensure feathers of a consistently high quality are grown [50]. Indeed, it could be hypothesized that the high cost of molt is not primarily due to the quantity of nutrients/protein directly needed for feather synthesis, but instead represents the cost of ensuring a constant supply of these materials during feather replacement, even when confronting immune challenges. There is experimental evidence that protein turnover during molt is 3.5 times that expected for peak rates of feather synthesis in white-crowned sparrows [51]. Given the consistent relation between rates of protein turnover and metabolism in endotherms of very different sizes [52], if molt provokes similar proportionate increases in protein turnover in all birds, it follows logically that molt costs will scale with metabolic intensity rather than the actual amount of feathers being replaced. Furthermore, such protein turnover may well follow a cyclic pattern, which would be in accordance with the metabolic profiles observed in molting WPHE and their lack of correspondence with feather growth (Figure 1). Additional indirect evidence for the high cost of such quality assurance mechanisms comes from studies of molting waterfowl. These birds characteristically show a brief period of simultaneous flight feather replacement, at a purportedly high cost when considering the relatively small mass of feathers being replaced [53]. Such costs may be necessary to ensure high quality, particularly for flight feathers. Unfortunately we did not compare the quality of feathers formed following plucking to those formed during natural molt.

**Table 3:** Published accounts of immune system alterations measured during the molt period.

Tissue	Change	Species	Reference
Spleen mass	increase	Willow tit	[54]
Monocytes	increase	House sparrow	[55]
		Red Knot	[47]
Total immunoglobulins	increase	King penguin	[56]
		Great tit	[57]
Humoral immunity	decrease	Red knot	[47]
		Domestic Chicken	[46]
Inflammatory response	decrease	House sparrow	[48]
		Red knot	[47]
		Domestic chicken	[46]

Regardless of the physiological mechanisms underpinning the metabolic costs entrained in the molt period, a key factor that modulates the physiological burden on the individual is the rate at which these costs are incurred [38], [42]. Decreased rates of molt should decrease the daily costs to an individual while also minimizing any potential disparity between resource requirements and the availability of these substrates in the diet. As such, relative molt costs, in the form of average daily costs as a proportion of pre-molt BMR, provide a more appropriate conceptual framework when considering both the physiological burden placed on an individual, and how this may differ between species. Molt duration appears to contract with increasing latitude in parallel with the decreasing summer duration. For example, bluethroats breeding and molting in the arctic undergo a complete molt in 62 days, practically twice as fast as East African stonechats (123 d), and almost three times as fast as WPHE (168 d, Table S1). Consequently, although the total cost of molt in WPHE is differs little from same-sized birds with much faster molt rates, their protracted molt period substantially reduces these molt costs on a daily basis to an extent that their daily molt costs are the lowest for all species studied. Interestingly, their prolonged molt appears to be mediated through a direct decrease in the rate at which each feather is grown, rather than a reduced frequency of feather shedding compared to north-temperate species [38]. These patterns are particularly beneficial for species living in environments with unpredictable resource availability, where breeding and molt schedules are likely to overlap.

## Supporting Information

Table S1 **Estimates of feather production cost based on indirect calorimetry for different species of birds.**
(DOCX)

Table S2 **Evaluation of evolutionary models for the efficiency of feather production.**
(DOCX)

Table S3 **Evaluation of null evolutionary models for the average daily cost of molt.**
(DOCX)
