# Supplementary material for: Inexplicable Inefficiency of Avian Molt? Insights from an Opportunistically Breeding Arid-Zone Species, Lichenostomus penicillatus
Source: PLoS One. 2011 Feb 2;6(2):e16230. doi: 10.1371/journal.pone.0016230 (PMC3032729; doi:10.1371/journal.pone.0016230)
Supplement: Table S1 — Estimates of feather production cost based on indirect calorimetry for different species of birds. (DOCX) [file pone.0016230.s001.docx]

Table S1. Estimates of feather production cost based on indirect calorimetry for different species of birds.

| *Species* | *Body mass (g)* | *BMR (kJ/d)* | *BMR_m_ (kJ/g/d)* | *Feather mass (g)* | *Molt duration (d)* | *Molt cost^1^ (kJ/g_feathers_)* | *Efficiency^2^ (%)* | *Daily increase^3^ (%)* | *Reference* |
| --- | --- | --- | --- | --- | --- | --- | --- | --- | --- |
|  |  |  |  |  |  |  |  |  |  |
| long-eared owl *Asio otus* | 280 | 106.0 | 0.38 | 31.6 | 134 | 116 | 22.7 | 25.8 | Wijnandts 1984 |
| kookaburra *Dacelo novaeguineae* | 340 | 98.2 | 0.29 | 22.9 | 120 | 69 | 38.0 | 13.4 | Buttemer *et al.* 2003 |
| European kestrel *Falco tinnunculus* | 210 | 77.8 | 0.37 | 20.1 | 180 | 132 | 19.9 | 18.9 | Dietz *et al.* 1992 |
| white-plumed honeyeater *Lichenostomus penicillatus* | 19 | 23.0 | 1.21 | 1.3 | 168 | 381 | 6.9 | 12.8 | This study |
| bluethroat *Luscinia s. svecica* | 17 | 39.7 | 2.34 | 1.2 | 62 | 862 | 3.1 | 42.0 | Lindström *et al.* 1993 |
| European stonechat *Saxicola torquata rubicula* | 15 | 24.7 | 1.65 | 0.9 | 123 | 580 | 4.6 | 17.2 | Klaassen 1995 |
| East African stonechat *Saxicola torquata axillaries* | 17 | 22.1 | 1.30 | 1.2 | 105 | 347 | 7.6 | 17.9 | Klaassen 1995 |
| white-crowned sparrow *Zonotrichia leucophrys gambelii* | 25 | 40.5 | 1.62 | 1.7 | 60 | 514 | 5.1 | 36.0 | Chilgren (in King 1981) |
| chaffinch *Fringilla coelebs* | 20 | 33.5 | 1.68 | 1.4 | 70 | 444 | 5.9 | 26.5 | Dolnik and Gavrilov 1979 |
| redpoll *Carduelis f. flammea* | 13 | 26.8 | 2.06 | 1.1 | 87 | 709 | 3.7 | 33.4 | Lindström *et al.* 1993 |

^1^Feather production costs represent the sum of molt-related increases in metabolic rate and the energy content of feathers (26.4 kJ.g^-1^ dry feathers; Murphy 1996).

^2^Feather production efficiency is the energy content of the feathers produced as a percentage of total production costs.

^3^Daily increase represents the average daily cost of molt (total energetic cost divided by the duration of feather replacement), as a percentage of pre-molt BMR.

**References**

Buttemer, W. A., *et al.* 2003. Thermoenergetics of pre-moulting and moutling kookaburras (*Dacelo novaeguineae*): they're laughing. J. Comp. Physiol. B 173: 223-230.

Dietz, M. W., *et al.* 1992. Energy requirements for molt in the Kestrel *Falco tinnunculus*. Physiol. Zool.65: 1217-1235.

Dolnik, V. R. and Gavrilov, V. M. 1979. Bioenergetics of molt in the Chaffinch (*Fringilla coelebs*). Auk 96: 253-264.

King, J. R. 1981. Energetics of avian moult. In: Norhring, R. (ed.) Acta XVII Congressus Internationalis Ornithologici. Verlag der Deutschen Ornithologen Gesellschaft, pp. 312-317.

Klaassen, M. 1995. Moult and basal metabolic costs in males of two species of stonechats: the European *Saxicola torquata ribicula* and the East African *S. t. axillaris.* Oecologia 104: 424-432.

Lindström, A., *et al.* 1993. The energetic cost of feather synthesis in proportional to basal metabolic rate. Physiol. Zool. 66: 490-510.

Wijandts, H. 1984. Ecological energetics of the long-eared owl (*Asio otus*). Ardea 72: 1-92
