# Supplementary material for: Inexplicable Inefficiency of Avian Molt? Insights from an Opportunistically Breeding Arid-Zone Species, Lichenostomus penicillatus
Source: PLoS One. 2011 Feb 2;6(2):e16230. doi: 10.1371/journal.pone.0016230 (PMC3032729; doi:10.1371/journal.pone.0016230)
Supplement: Table S2 — Evaluation of evolutionary models for the efficiency of feather production. (DOCX) [file pone.0016230.s002.docx]

Table S2. Evaluation of evolutionary models for the efficiency of feather production.

| *model* | *K* | *n* | *LgL* | *AICc* | *ΔAICc* | *LΔAICc* | *AICcW* | *Evidence ratio* | *r^2^* | *d/ λ* |
| --- | --- | --- | --- | --- | --- | --- | --- | --- | --- | --- |
| *BMRm model* | | | | | | | | | | |
| OU | 4 | 10 | 10.906 | -5.813 | 0.000 | 1.000 | 0.551 | 1.000 | 0.884 | 1.05 |
| Pagel’s λ | 4 | 10 | 11.258 | -6.515 | 0.702 | 0.704 | 0.388 | 1.421 | 0.884 | 0.92 |
| OLS | 3 | 10 | 10.702 | -11.404 | 5.591 | 0.061 | 0.034 | 16.374 | 0.944 |  |
| PGLS | 3 | 10 | 10.899 | -11.798 | 5.985 | 0.050 | 0.028 | 19.933 | 0.886 |  |
| *Evolutionary (null) model* | | | | | | | | | | |
| PGLS | 2 | 10 | 0.063 | 5.587 | 0.0000 | 1.000 | 0.729 | 1.000 | 0 |  |
| Pagel’s λ | 3 | 10 | 0.465 | 9.071 | 3.4836 | 0.175 | 0.128 | 5.708 | 0 | 0.93 |
| OU | 3 | 10 | 0.127 | 9.747 | 4.1592 | 0.125 | 0.091 | 8.001 | 0 | 1.14 |
| OLS | 2 | 10 | -3.699 | 13.112 | 7.5246 | 0.023 | 0.017 | 43.047 | 0 |  |

Evaluation of evolutionary models for the efficiency of feather production (log_10_), including ordinary least squares (OLS, no phylogenetic signal), phylogenetic generalized least squares (PGLS, Brownian motion), Ornstein–Uhlenbeck process (OU, drift about a fitness peak), or Pagel’s *λ* (branch lengths transformed using Pagel’s *λ* parameter). Models with a phylogenetic signal were most parsimonious, and together with d/*λ* values approximately equal to one, indicate a strong phylogenetic signal in conversion efficiency during molt. Evidence for such phylogenetic patterns are, however, severly hindered by the lack of data from small (high BMRm) non-passerines and large (low BMRm) passerines.
