# Supplementary material for: Inexplicable Inefficiency of Avian Molt? Insights from an Opportunistically Breeding Arid-Zone Species, Lichenostomus penicillatus
Source: PLoS One. 2011 Feb 2;6(2):e16230. doi: 10.1371/journal.pone.0016230 (PMC3032729; doi:10.1371/journal.pone.0016230)
Supplement: Table S3 — Evaluation of null evolutionary models for the average daily cost of molt. (DOCX) [file pone.0016230.s003.docx]

Table S3. Evaluation of null evolutionary models for the average daily cost of molt.

| *model* | *K* | *n* | *LgL* | *AICc* | *ΔAICc* | *LΔAICc* | *AICcW* | *Evidence ratio* | *r^2^* | *d/ λ* |
| --- | --- | --- | --- | --- | --- | --- | --- | --- | --- | --- |
| *Evolutionary (null) model* | | | | | | | | | | |
| OLS | 2 | 10 | -36.749 | 79.213 | 0.000 | 1.000 | 0.698 | 1.000 | 0 |  |
| Pagel’s λ | 3 | 10 | -36.225 | 82.450 | 3.237 | 0.198 | 0.138 | 5.046 | 0 | 0.004 |
| OU | 3 | 10 | -36.714 | 83.429 | 4.216 | 0.122 | 0.085 | 8.231 | 0 |  |
| PGLS | 2 | 10 | -38.927 | 83.569 | 4.356 | 0.113 | 0.079 | 8.830 | 0 | 0.006 |

Evaluation of null evolutionary models for the average daily cost of molt (% pre-molt BMR), including ordinary least squares (OLS, no phylogenetic signal), phylogenetic generalized least squares (PGLS, Brownian motion), Ornstein–Uhlenbeck process (OU, drift about a fitness peak), or Pagel’s *λ* (branch lengths transformed using Pagel’s *λ* parameter).
